# Supplementary material for: Ursolic and Oleanolic Acids: Plant Metabolites with Neuroprotective Potential
Source: Int J Mol Sci. 2021 Apr 27;22(9):4599. doi: 10.3390/ijms22094599 (PMC8124962; doi:10.3390/ijms22094599)
Supplement: Supplementary file 1 [file ijms-22-04599-s001.zip › ijms-1184126-supplementary.pdf]

Supplementary data

Gudoityte et al. (2021)

Various experimental models (see Table 2 in the main text) have shown that single dose or prolonged administration of ursolic acid (UA) might have a positive effect on mitochondria. Therefore, we analysed the effects of UA on isolated mouse mitochondria respiration rates at different metabolic states.

For this purpose we used two different schemes. Mitochondrial Leak respiration (Figure S1), in the presence of 5 mM pyruvate and 2 mM malate as substrates, was stepwise titrated with UA (separate additions of 1 μM). Identical scheme was used for the vehicle, i.e. Leak respiration was stepwise titrated with dimethyl sulfoxide (DMSO) 0.025%. Control Leak respiration was recorded in the presence of pyruvate and malate only. It was found that UA had no effect on the isolated mouse brain mitochondrial Leak respiration as compared with Control, whereas 0.2-0.25% of DMSO increased it.

In addition, we evaluated the effects of 5 μM UA and 0.125% DMSO on mitochondrial respiration at different metabolic states (Figure S2). Both substances had no statistically significant effects on the respiration rates as compared with Control.

Thus, we found that UA had no effect on isolated mouse brain mitochondrial respiration rates. However, we cannot rule out the possibility that UA indirectly affects brain tissue mitochondria acting through certain intracellular targets (see Table 2).

Isolation of mitochondria

Mouse brain mitochondria were homogenised in the isolation medium (IM) containing 225 mM mannitol, 75 mM sucrose, 10 mM MOPS, 1 mM EDTA, 0.1 % BSA, and 1 mM EGTA (pH 7.2). The homogenate was centrifuged at 1000 ×g for 5 min (4°C), and the collected supernatant at 10 000 ×g for 10 min (4°C). The obtained pellet was suspended in IM and centrifuged for 10 min at 10 000 ×g (4°C). The final mitochondrial pellet was suspended in IM, and the Biuret method was used for the mitochondrial protein determination.

Respirometric measurements

The oxygen uptake rates of isolated mitochondria were recorded using a respirometer Oxygraph-2k (Oroboros Instruments, Innsbruck, Austria) at 37°C in the measurement medium MIR5 containing 110 mM Sucrose, 20 mM HEPES, 60 mM Lactobionic acid, 20 mM Taurine, 3 mM $MgCl_2$, 0.5 mM EGTA, 10 mM KH_2PO_4 (pH 7.2), and 0.25 mg/1 ml mitochondrial protein. Mitochondrial respiration rates were expressed as pmol O/s/mitochondrial protein. Results are presented as means ± S.E.

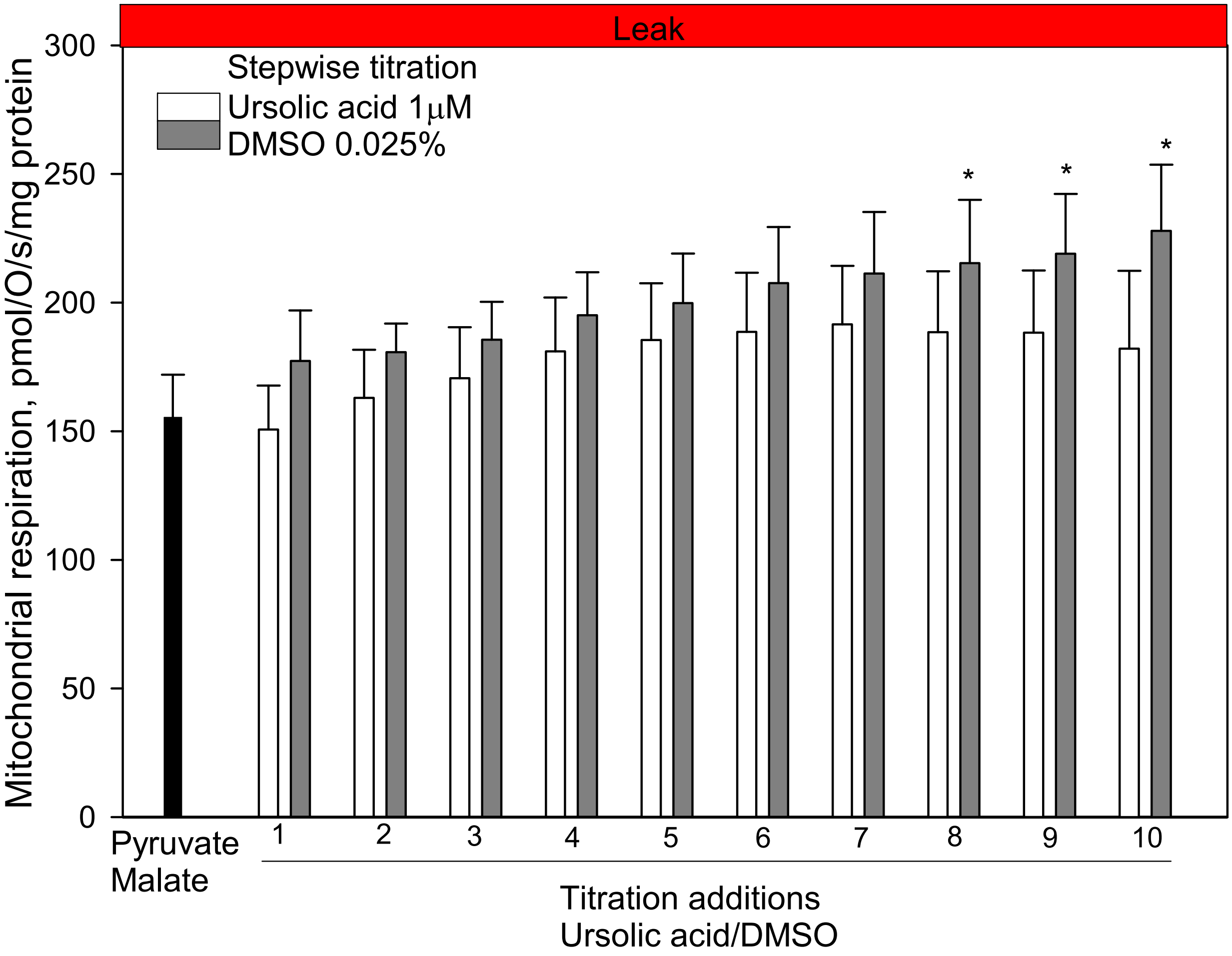

Figure S1. Effect of ursolic acid on Leak respiration. Mitochondrial respiration in the presence of pyruvate and malate was stepwise titrated with 1 μM UA (UA concentration after the 1st addition was 1 μM, whereas after the last 10th addition reached 10 μM) or with DMSO as vehicle (0.025% concentration after the 1st addition and 0.25% after the last addition).

* - statistically significant difference when compared with control group; ANOVA with Post hoc Tukey test, $p<0.05$, n=3-5.

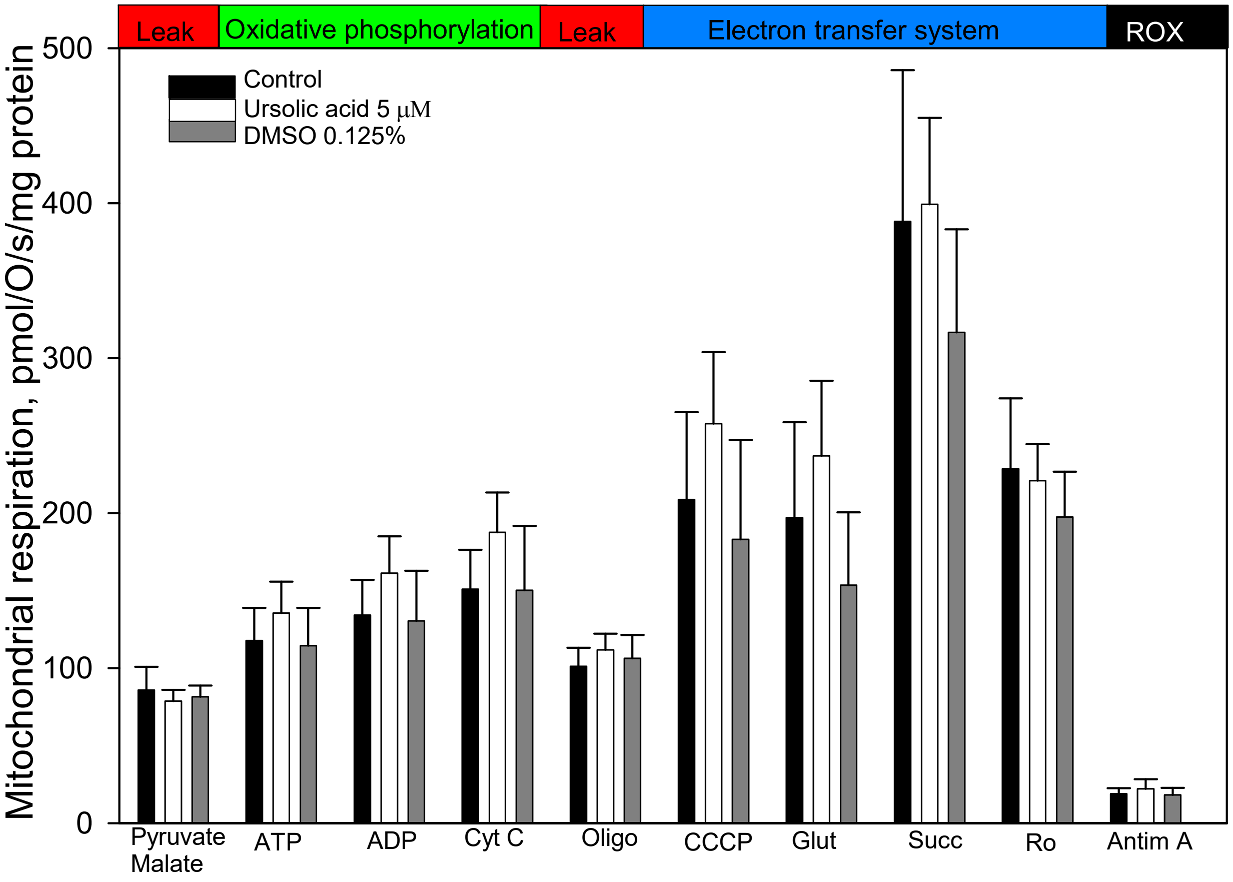

Figure S2. Effects of 5 μM ursolic acid on mouse brain mitochondrial respiration at different metabolic states. At the beginning the Leak respiration rate with isolated mitochondria in the presence of pyruvate (5 mM) and malate (2 mM) that stimulate complex I was measured. Oxidative phosphorylation was stimulated by adding 1 mM ATP and 2.5 mM ADP, followed by cytochrome c (Cyt c, 0.01 mM). Oligomycin inhibited the ATP-synthase and returned the electron transport chain to the Leak state. Whereas subsequently added uncoupler carbonyl cyanide 3-chlorophenylhydrazone (CCCP) stimulated the electron transfer system, whose activity was sustained by additional substrates: glutamate (Glut; complex I) and succinate (in the presence of rotenone (Ro); complex II). Antimycin A (Antim A) inhibited the complex III and enabled to estimate the residual oxygen comsumption (ROX). ANOVA with Post hoc Tukey test ($p<0.05$) was used for the statistical analysis. n=3-5.
